# Facilitators and barriers to implementing electronic referral and/or consultation systems: a qualitative study of 16 health organizations

**DOI:** 10.1186/s12913-015-1233-1

**Published:** 2015-12-19

**Authors:** Delphine S. Tuot, Kiren Leeds, Elizabeth J. Murphy, Urmimala Sarkar, Courtney R. Lyles, Tekeshe Mekonnen, Alice Hm Chen

**Affiliations:** Division of Nephrology at San Francisco General Hospital, University of California, San Francisco, San Francisco, CA 94110 USA; Center for Innovation in Access and Quality at San Francisco General Hospital, University of California, San Francisco, San Francisco, CA 94110 USA; Division of Endocrinology at San Francisco General Hospital, University of California, San Francisco, San Francisco, CA 94110 USA; Division of General Internal Medicine at San Francisco General Hospital, University of California, San Francisco, San Francisco, CA 94110 USA

**Keywords:** Electronic referral, Electronic consultation, Access to care, Health technology, Specialty care, Implementation, Health system redesign

## Abstract

**Background:**

Access to specialty care remains a challenge for primary care providers and patients. Implementation of electronic referral and/or consultation (eCR) systems provides an opportunity for innovations in the delivery of specialty care. We conducted key informant interviews to identify drivers, facilitators, barriers and evaluation metrics of diverse eCR systems to inform widespread implementation of this model of specialty care delivery.

**Methods:**

Interviews were conducted with leaders of 16 diverse health care delivery organizations between January 2013 and April 2014. A limited snowball sampling approach was used for recruitment. Content analysis was used to examine key informant interview transcripts.

**Results:**

Electronic referral systems, which provide referral management and triage by specialists, were developed to enhance tracking and operational efficiency. Electronic consultation systems, which encourage bi-directional communication between primary care and specialist providers facilitating longitudinal virtual co-management, were developed to improve access to specialty expertise. Integrated eCR systems leverage both functionalities to enhance the delivery of coordinated, specialty care at the population level. Elements of successful eCR system implementation included executive and clinician leadership, established funding models for specialist clinician reimbursement, and a commitment to optimizing clinician workflows.

**Conclusions:**

eCR systems have great potential to streamline access to and enhance the coordination of specialty care delivery. While different eCR models help solve different organizational challenges, all require institutional investments for successful implementation, such as funding for program management, leadership and clinician incentives.

**Electronic supplementary material:**

The online version of this article (doi:10.1186/s12913-015-1233-1) contains supplementary material, which is available to authorized users.

## Background

Suboptimal delivery of specialty care is one of the most pressing issues facing our health care system today. Studies of diverse health delivery organizations have documented poor access to specialty care, with wait times as high as 6–12 months in some communities [1–3], and highly prevalent gaps in coordination and inter-provider communication. The current primary-specialty care interface results in avoidable specialist visits, duplicate testing, and delayed diagnoses [4]. In turn, this leads to inefficient use of scarce specialty resources [5], preventable harm to patients, and unnecessary costs. In the United States, passage of the Affordable Care Act is anticipated to increase demand for specialty care. New models for providing access to timely, coordinated, cost-effective specialty care are needed. Widespread adoption of electronic medical records (EMR) has fostered a growing interest in the development of electronic referral and/or consultation (eCR) systems to enhance communication among providers as well as streamline access to and improve coordination of specialty care delivery.

In 2007, San Francisco General Hospital (SFGH) implemented eReferral, a home-grown integrated eCR system that has enhanced access to and the delivery of coordinated specialty care while achieving high levels of satisfaction among primary care providers (PCPs) and specialists [6, 7]. With this eCR, specialist clinicians review each referral and use the system to schedule a routine or expedited clinic visit, recommend additional diagnostic evaluation before scheduling a clinic visit (pre-consultation exchange), or provide education and management without a visit (virtual co-management). Since its implementation, we have received inquiries from a wide range of organizations with differing payment structures interested in adopting the eReferral model. Because eCR systems result in new responsibilities for both PCPs and specialists, creating such a system is disruptive to the traditional specialty care model and thus challenging to implement. These challenges have been documented by individual organizations in Australia [8] and Canada [9] but a more comprehensive examination of implementation challenges is lacking [10]. To better understand the drivers, facilitators and barriers to adopting eCR platforms across diverse health care delivery systems in the United States, as well as to garner best practices, we conducted key informant interviews with leaders of health delivery organizations who had expressed interest in implementing an eCR system.

## Methods

### Design and sampling

The study was approved by the Committee on Human Research at the University of California, San Francisco. Using a limited snowball sampling approach, we contacted leaders of 22 health delivery organizations who had previously contacted SFGH to learn about its eCR system. Represented organizations were diverse, including academic medical centers, health plans, public health care delivery systems and community health networks, and were at different stages of eCR implementation at the time of contact, ranging from pilot projects to full expansion. Email invitations to participate in key informant interviews about each organization’s progress towards adopting an electronic referral and/or consultation system were sent between January 2013 and April 2014. Those interviewees referred us to leaders of two other organizations who we subsequently contacted. Of the 24 health care delivery organizations contacted, two were international and were excluded from the analysis given their very different financial structures. We did not continue to sample for additional interviewees after April 2014 when we determined that no new information was emerging from the interviews and thematic saturation had been reached.

### Definitions

In the United States, there are no standard definitions for electronic referrals or electronic consultations. Per the Affordable Care Act, a referral is a written order from a primary care doctor for a patient to see a specialist or get certain medical services [11]. Consultations are not clearly defined by the healthcare.gov website; however, they are generally considered to reflect communication between clinicians about general or patient-specific questions [12]. We build upon these ideas and define an *electronic referral* as a technology-enabled structured request by a referring provider, most of often a PCP, to a specialist with an expectation that the patient will be seen in person by the specialist. Electronic referral systems provide an efficient mechanism for referral management, tracking, and, if reviewed by specialists, triage. An *electronic consultation* is defined as a request by a provider for a patient’s condition and treatment to be evaluated by a specialist with management advice if appropriate; it does not carry the expectation that a specialist will see the patient. While both electronic referral and electronic consultation systems allow specialists to participate in pre-consultative exchange to ensure adequate diagnostic workup prior to an in-person visit, only electronic consultation systems encourage ongoing, bi-directional communication between primary care and specialist providers. This functionality promotes virtual co-management, whereby specialists provide longitudinal guidance to PCPs for patient management without the need for an in-person patient evaluation. Electronic referral systems may be used in parallel to electronic consultation systems, with different portals for referring providers. Integrated eCR systems, such as SFGH’s eReferral, have a single portal of entry for the referring provider and do not require providers to distinguish referrals from consultations. These systems rely upon specialist review of every request for specialty expertise, thereby leveraging the functionalities of both electronic referral and electronic consultation systems for the delivery of specialty care.

### Data sources

Phone interviews were conducted by two investigators (KL and DST). Audio-recorded interviews consisted of nine questions that covered the following topics: drivers for implementing an electronic referral, consult, or integrated eCR system, facilitators and barriers to implementation, evaluation metrics, and key lessons learned (Additional file 1: Table S1). Each conversation lasted 30–60 min and was professionally transcribed. Informed consent was obtained by all participants.

### Analysis

We analyzed interview transcripts using directed content analysis [13]. We developed a list of previously published factors that influence eCR adoption and use [8] prior to the first interview (Additional file 1: Table S2). This deductive coding scheme allowed us to apply four major categories for the analysis: drivers (which included the primary reason during the planning phase for pursuing eCR systems), barriers and facilitators to the actual implementation process, and evaluation metrics for understanding how and when eCR systems were effective. Then, during the analysis, we used open coding within each of these four categories to describe the emerging ideas from respondents, and to compare how the themes differed by type of eCR being implemented and organizational type. Two investigators (KL and DST) coded the first few transcripts separately to determine themes, ensuring that these transcripts included all types of healthcare delivery systems in the sample (community health network, county public system, academic medical center, and health plan). The entire coding framework was shared with all co-authors and agreed upon after analyzing the first six transcripts.

## Results

### Participating organizations

Of the 22 organizations contacted in the United States, 16 (73 %) agreed to participate. Participating organizations included academic medical centers (*n* = 4), health plans (*n* = 2), public health care delivery systems (*n* = 7), community health networks (*n* = 2), and other nonprofit organizations (*n* = 1). Organizational size varied considerably, ranging from 400 to 1,300,000 patients served annually, as did the number of patients actually served by each eCR system (range 47 to 100,000 (Table 1)).

**Table 1:** Characteristics of participating health delivery organizations, ordered by type of eCR system. SFGH included as reference

Geographic Area	Type of Organization	Safety net	Patients Served Annually	Type of electronic referral and consultation system (eCR)	Number of patients with electronic referrals or consultations submitted
Colorado	Public system – county	Yes	133,000	None	none; pre-pilot
Washington	Public system – county	Yes	240,000	None	none; pre-pilot
Hawaii, all islands	Health plan	No	314,500	Referral	100 patients in referral pilot as of October 2013
Central Massachusetts	Academic medical center	No	1,000,000	Referral	Appointment requests for 300 patients as of March 2014
Southern California	Public system – county	Yes	500,000	Referral	18,000 consults in 2013
Southern California	Public system – county	Yes	240,000	Referral	59,400 in 2013
Northern California	Network of community health centers	Yes	31,000	Referral	4000 patients with consults
Boston, Massachusetts	Academic medical center	No	data not available	Consultation	47 consults in 3-month pilot as of October 2013
Southern California	Academic medical center	No	971,000	Consultation	330 consults
Northern California	Academic medical center	No	232,774	Consultation	550 consults between April 2012 and May 2013
Southern California	Advocacy organization	Yes	400	Consultation	250 patients with consults in 2012
Northern California	Public system – county	Yes	100,000	Consultation	data not available; significant volume
Northern California	Public system – county	Yes	80,000	Consultation	data not available
Connecticut	Network of community health centers	Yes	130,000	Integrated eCR	125 consults for 120 patients
Southern California	Health plan	Yes	1,300,000	Integrated eCR	100,000 consults
Southern California	Public system – county	Yes	850,000	Integrated eCR	60,000 consults
San Francisco General Hospital	Public system – County	Yes	123,500	Integrated eCR	58,000 yearly

Six organizations did not participate. Leaders of two of the organizations replied to initial emails inquiring about their eCR platforms, but interviews were not scheduled due to logistical difficulties. Of those two, one has implemented an electronic referral system and the other has an electronic scheduling system. The status of the other four organizations’ eCR systems are unknown, as organizational representatives did not respond to multiple email inquiries and invitations to schedule a key informant interview. We could find no evidence from literature and web searches that they have implemented an eCR.

### Description and functionalities of electronic referral, consultation, and integrated eCR systems

#### Electronic referral

Of participating organizations, five had either piloted (*n* = 1) or implemented (*n* = 4) an electronic referral system (Additional file 1: Table S3a). These included relatively small programs, including one health plan and one academic medical center, and three larger programs implemented by safety-net institutions, entities that provide a patchwork of services to the uninsured, under-insured and indigent populations who would otherwise have little access to health care services. Organizations relied on different IT platforms: three were integrated into the EMR and two were standalone systems. All systems had the same core functionality of enabling PCPs and clinic coordinators to track referrals. PCPs, not clinic coordinators, were expected to submit the initial referral, which consisted of either a structured template (*n* = 2) or a free text form (*n* = 3). Those with referral templates also had referral guidelines embedded in their electronic workflow to help PCPs complete medical workups prior to referring to specialty care. In all systems, administrative staff reviewed referral requests and distributed them to the appropriate specialist. Specialists engaged in pre-consultative exchange in only one system.

#### Electronic consultation

Six systems developed electronic consultation platforms, including three small systems used by academic medical centers and three larger systems implemented by safety-net organizations: one small advocacy organization and two county public healthcare delivery systems (Additional file 1: Table S3b). While the platform used by the advocacy organization is no longer active, the remaining five systems have been implemented for a varying number of specialties, ranging from 12 to 39. Several different IT platforms were used: three were integrated into the EMR and the others were standalone web-based systems. All electronic consultation systems enabled bi-directional communication between referring and specialty providers. Referrals were submitted by either the PCP (*n* = 5) or a referral coordinator (*n* = 1). In five of the six systems, reports/studies or lab results could be appended to the consultation request. By reviewing the consult question and appended data when applicable, specialists in each system could identify a subgroup of patients that did not need a face-to-face visit and would benefit from electronic consultation alone. As with the electronic referral systems, organizations that provided referral guidelines (*n* = 2) also required their PCPs to use specific referral templates.

### Integrated eCR systems

Three organizations implemented integrated eCR systems similar to SFGH’s eReferral, all of which served safety-net populations (Additional file 1: Table S3c). The eCR platform was embedded into the EMR for only one system. All integrated eCRs had the same core functionalities of referral management/tracking and the possibility of bi-directional communication for all referrals. PCPs submitted electronic referrals to designated specialist reviewers who determined whether a patient needed to be seen for a face-to-face visit or could be managed via electronic consultation alone. The smallest organization did not have referral guidelines or templates, while the other two organizations did.

### Drivers for implementing electronic referral, consultation, or integrated eCR systems

While many leaders expressed similar challenges regarding the delivery of specialty care, they cited different reasons for implementing electronic referral versus electronic consultation or integrated eCR systems. Electronic referral systems were primarily implemented to enhance operational and clinical efficiency (Table 2). Prior referral methods, which relied on paper and fax, contributed to inefficient in-person specialty visits because of inadequate workup and illegible communication between primary care and specialty care providers. In turn, this led to poor specialist satisfaction.

**Table 2:** Main drivers, facilitators and barriers to implementation of referral, consultation and integrated eCRs and representative quotes

Drivers		Representative quotes
Referral systems	Enhance operational and clinical efficiency	“Specialists wanted to have all of the relevant clinical history for patients prior to a visit; productive visits are key and prior communication [wasn’t] sufficient.”
Consultation systems and Integrated eCRs	Poor access to specialty care	“The service was developed to improve access to high-need specialties with long wait times.”
	Leakage of patients to other systems	“Drivers included coordinating and improving integration of care with the goal of retaining patients; payor data showed that approximately 30 % of patients were going outside of [organization’s name] for specialty care.”
	Enhance care coordination and communication	“We’re trying to build good relationships with our community clinics to create an integrated safety net care system.” “I think efficiency helps the supply–demand mismatch… we have driven down the mismatch with eConsult, not by inventing new specialists, but by using our existing specialists in a better way.”
Facilitators		Representative quotes
Referral systems	Engaged executive leadership	“For our program, it was important to make eConsults/eReferrals mandatory [by the leadership]. We found that [others] that did not do this had low uptake.”
	Early clinician adopters	“Having a physician leader who was able to have dedicated time to have lots of the conversations with people, to message it, to overcome concerns and resistance, to really be the driving force behind it, I think was critical in our implementation.”
Consultation systems and Integrated eCRs	Provider incentives	“[Our system] is now funding reimbursement of specialists’ time for using the service.”
	User-friendly technology	“The template was easy to build and make friendly for staff and the doctors.”
	Platform integrated into electronic medical record	“We have a platform available within our electronic health record program that we were able to adapt to our need/s.”
Barriers		Representative quotes
Referral systems	Provider resistance to change workflows	“As you well know, unless you can mandate, it is very difficult to get PCPs to adapt if they view this as taking any more time.”
	Lack of eCR integration into electronic medical record	“With no shared [technology], it has been difficult to get providers to [move past the workflow issues] and see the benefit of … improved integration of care.”
Consultation systems or integrated eCRs	Primary care provider resistance to change in workflows	“Many physicians didn’t want to submit [the referral] themselves.”
	Lack of reimbursement mechanisms	“The biggest barrier to adoption we faced was reimbursement. … It is this funding issue that is preventing expansion of the program to include additional specialties.” “In order to support adoption of the electronic consult system, we obtained grant funding. We are currently using the results … to build a case for the state reimbursing electronic referrals.”
	Specialist provider liability concerns	“When we implemented … we got quite a lot – not surprising, but consistent – feedback or questions or skepticism from specialists with the liability, specialist skepticism about whether the PCP [would] be able to provide reliable information.”

By contrast, the main driver of implementing electronic consultation or integrated eCR systems was poor access to specialty care, particularly evidenced by long patient wait times for in-person specialty appointments. Not only were these wait times vexing for health system leaders concerned about the delivery of timely, coordinated specialty care, but they were also thought to contribute substantially to poor patient satisfaction scores and to negatively impact revenue because of leakage of patients to neighboring systems with quicker access to specialty care appointments. Less prevalent reasons driving adoption of electronic consultation or integrated eCR systems included the desire to leverage existing specialty capacity to care for a larger number of patients and to enhance communication among referring and consulting providers. By facilitating virtual co-management, electronic consultation systems were expected to enhance primary care capacity to longitudinally manage conditions with specialist support as needed.

### Facilitators for implementing electronic referral, consultation, or integrated eCR systems

Organizations that achieved high levels of sustained provider participation in their eCR systems cited engaged leadership as the most important facilitator (Table 2). In most circumstances, leaders included a combination of high-level executives, such as Chief Medical Officers, as well as physician leaders who were early adopters. Executive leaders prioritized implementation of the program in response to specific organizational challenges and used organizational priorities, such as operational efficiency, to drive adoption. The physician leaders, by contrast, served as clinical champions, increasing uptake among colleagues through modeling. Existing collegial relationships with specialists were also deemed important by all interviewees.

Leaders from organizations that implemented electronic consultation or integrated eCR systems also emphasized the importance of provider incentives and reimbursement mechanisms. In many cases, PCPs were given productivity “credit” or access to referral managers to expedite the referral process and specialists were paid to perform pre-consultative exchange and virtual co-management. Of interest, leaders of organizations whose electronic consultation systems had been in place for many years had started thinking about ways to incentivize quality of electronic communication. Other facilitators included having user-friendly technology, as well as technology that could be integrated into the organization’s EMR and embedded into existing workflows with dedicated program support staff to perform outreach.

### Barriers to implementing electronic referral, consultation or integrated eCR systems

All organizations encountered challenges. Resistance to change, particularly to changes in PCP workflow, emerged prominently during our interviews (Table 2). Without exception, with every eCR, PCP workload increased, as they were expected to navigate new technology to enter a referral question and pertinent patient data. Additionally, PCPs acquired extra work in managing conditions that they used to refer. Specialists also experienced greater workload in the form of pre-consultative exchange and virtual management, which also served as a barrier to implementation. Working with standalone eCR systems that were not integrated with existing EMRs was also a challenge shared by many organizations, which resulted in duplication of work for providers and/or staff.

The most oft-cited barrier to widespread implementation of electronic consultation or integrated eCR systems was a lack of resources. Specifically, lack of reimbursement mechanisms for specialists, inadequate funding for on-going costs to support the technology platform, and a dearth of administrative support to maintain the system were mentioned by many study participants (Table 2). Another challenge unique to electronic consultation and integrated eCR systems but not referral systems was specialist concern about liability.

### Evaluation metrics

While combinations of drivers, facilitators and barriers to adoption of eCR systems may have differed among individual health care delivery systems, all organizational leaders expressed a commitment to program evaluation. Interviewees highlighted the importance of evaluation metrics for ongoing quality improvement activities to ensure that their system met organizational needs and enhanced the primary-specialty care interface. All systems, regardless of their functionality, had operational metrics in place from inception that included: referral volume, number and type of services available, number of referring primary care sites and providers, and time from referral to treatment or first visit. With respect to common scheduling and clinical metrics, organizations with all eCR types examined the number of days needed to confirm patient appointments, the clinical reason for consultations and PCP satisfaction.

Unique evaluation metrics pertinent to electronic referral, consultation, and integrated eCR systems are presented in Table 3. Many referral systems examined percentage of referrals completed electronically vs. fax/paper and patient disposition as determined by a referral management department, as well as unique scheduling metrics including the percentage of clinic template slots used for patients referred electronically rather than via paper or fax. By definition electronic systems did not include communication metrics. Health care systems that implemented electronic consultation or integrated eCRs often examined additional operational metrics related to specialty care access, such as time to first specialist response and patient disposition as determined by the specialist reviewer. Most systems did not yet have benchmark goals for these metrics, with the exception of time to specialist response, where the goal was typically 2–3 business days. Many organizations with consultation and integrated eCRs also examined communication quality. In particular, several organizations looked at the number of exchanges between a PCP and specialist per consult, PCP expectation with consultation requests, and quality of content in PCP referral and specialist response.

**Table 3:** Unique existing evaluation metrics pertinent to referral, consultation and Integrated eCR systems, by domain

	Operational	Scheduling	Clinical	Communication
Referral	• Percent of referrals completed electronically vs. fax/paper	• Percent of eReferral slots used	• Patient follow-up by electronic vs. paper/fax referral	
	• Disposition (scheduled vs not scheduled) as determined by referral management department	• Slot availability for eReferred-patients		
	• Wait time for specialty service by insurance status			
Consultation and integrated eCR	• Time to first specialist response		• Self-reported PCP ability to manage a patient with specialist guidance	• Specialist satisfaction
	• Disposition (e.g., scheduled immediately, scheduled after review, consultation only) as determined by specialist reviewer		• Emergency department utilization	• PCP expectation for referral
	• Time spent by specialist		• Cardiac outcomes: appropriate diagnoses, percent of patients with blood pressure control, PCP prescription of guideline-conoirdance cardiovascular medications^a^	• Quality of PCP referral
	• Economic impact of provider reimbursement strategy		• Specialty clinic complexity^a^	• Quality of specialist response
	• Patient leakage to other health systems for specialty care			• Number of exchanges per consult
	• Primary care clinic adoption of system^a^			• Number of consults with document uploads^a^

^a^Denotes metrics that were only examined among integrated eCR systems

As with traditional primary care-specialty care interactions, measuring the impact of eCR systems on patient outcomes and PCP/specialist capacity were frequently cited as the most clinically relevant but challenging metrics to capture. Many interviewees expressed the desire to measure whether their system had met patient needs, but determining whether the right patient received specialty expertise “by the right specialist at the right time, in the right way” remained elusive. To date, only one organization with an integrated eCR has measured true clinical outcomes, comparing PCP prescription of guideline-concordant cardiovascular medications among those who sent electronic consultations to cardiologists versus those who referred patients via usual methods (Table 3). Adverse cardiac outcomes were also compared among patients who were referred electronically versus traditional methods. Three organizations examined other types of clinical metrics that were not directly patient-facing, including specialty clinic complexity and impact on PCP capacity to manage “specialty problems”. Notably, none of the systems have been able to measure cost savings or calculate a “return on investment” for their eCR.

## Discussion

To date, the term “electronic referral system” has been used in the United States to describe information technology systems with a wide range of functionality and specialist involvement. Some eCR systems are standalone web-based programs, while others are fully embedded into EMRs. Some are purely referral tracking tools, without any specialist clinician involvement prior to an appointment, while others offer electronic consultation as a means of providing timely specialty expertise for patients with low complexity issues. A small number of organizations have implemented integrated eCR systems where a specialist reviews each referral, thereby maximizing opportunities for pre-consultative guidance and virtual co-management; these are most similar to SFGH’s eReferral system. While offering solutions to different organizational challenges, all eCR systems are actively changing the primary care-specialty care interface across diverse health care delivery settings.

We found that integrated eCRs have been implemented mainly in public health care systems that are responsible for a defined population of individuals and traditionally have had difficulties with specialty access. On the one hand, requiring specialty review of each referral request entails additional work for both PCPs and the specialist reviewers. This requires additional resources to compensate specialist reviewer time, and may prompt PCPs who are unable or unwilling to absorb the additional work of managing conditions they would usually refer, to seek alternate consultants. On the other hand, having a single entry point for all referrals to a given specialty provides a population health perspective for the system and better ensures that scheduled specialty visits are appropriate with complete pre-visit evaluations. In this manner, integrated eCRs enable health care organizations to act as stewards of scarce specialty resources and more effectively match supply of and demand for specialist expertise at a large scale, thereby maximizing population health [14]. That said, some Veterans Administration and Kaiser Permanente sites, two integrated health systems touted for their population-health management, have implemented eCRs that maintain separate electronic referral and consultation portals rather than an integrated eCR platform [15, 16]. It appears that these health systems encourage but do not mandate specialist review of all referral requests and do not compensate providers for this additional effort.

**Fig. 1:**
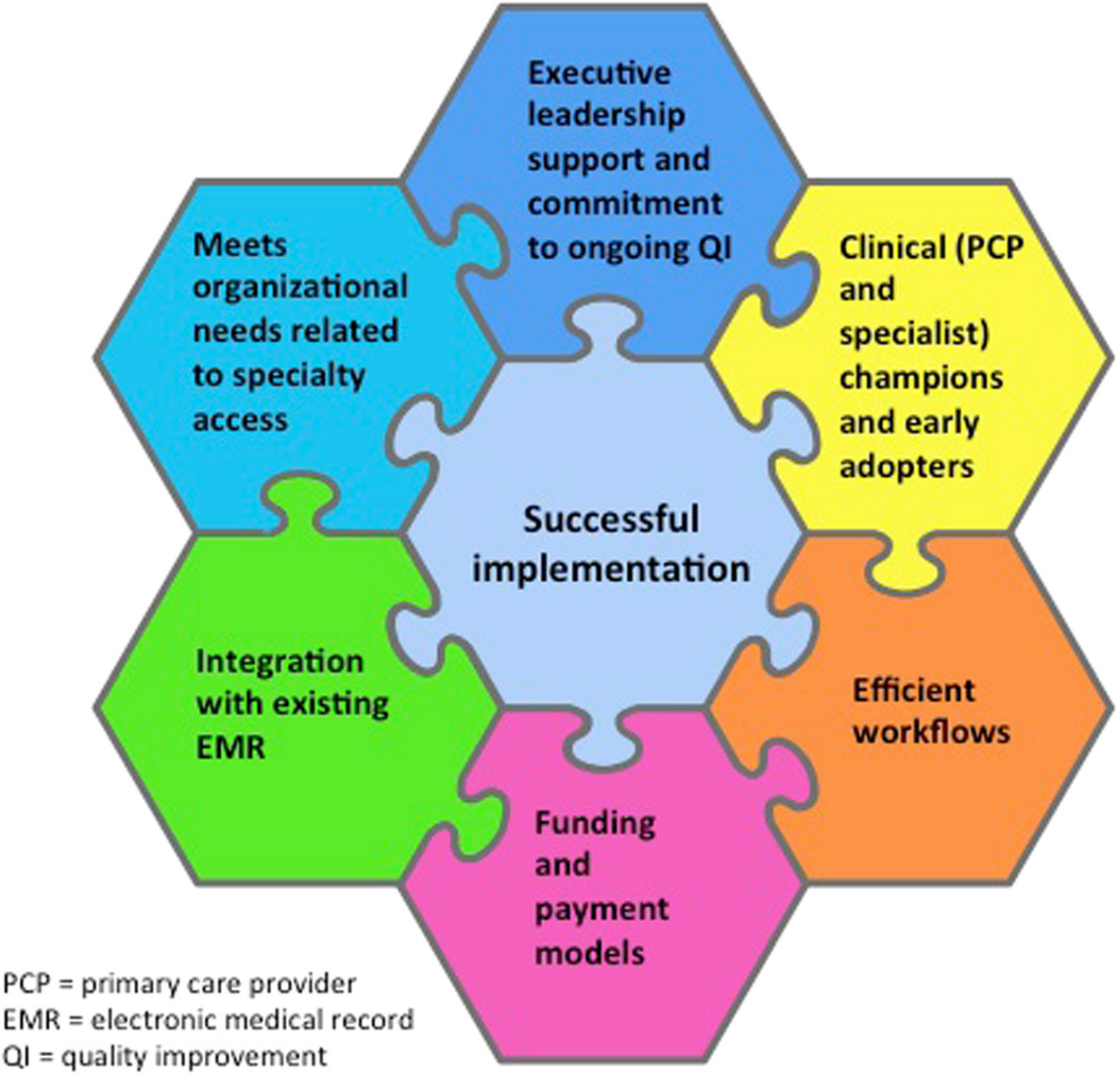
Elements of successful implementation of electronic consultation systems

Per our interviews across diverse health care models, eCR systems that have developed into sustainable, successful systems are those that provide both electronic referral and consultative activities, including those that maintain the two as separate entities, as well as those systems that have integrated them into a single portal of entry. The technical platform and workflow inherent to an electronic referral system are often prerequisites for electronic consultative activities, but they are not sufficient. The important elements for successful implementation of electronic consultation systems that emerged from our data include funding for clinician reimbursement as well as program management, a marketing plan with positive messaging to stakeholders, a commitment to create efficient clinician workflows via system re-design and dedication to on-going quality improvement in response to key evaluation metrics (Fig. 1). Integration of the consultative system with existing EMR platforms is also a facilitator for implementation, though it may not be absolutely necessary, given the success of some programs using standalone technologies. But, having a user-friendly, affordable eCR system is key for adoption and sustainability. Leaders of organizations that successfully implemented eCR systems also cultivated a collaborative approach, involving primary care and specialty care providers in the development of systems policies and referral guidelines to fully support stakeholder buy-in and end-user adoption. Many of the essential implementation elements in Fig. 1 are very similar to what has been previously cited for successful implementation of other health system changes in the United States, such as adoption of electronic health records [17], transformation of primary care practices into patient-centered medical homes [18], development of Accountable Care Organizations [19] and creation of care coordination agreements among primary care and specialty care providers [20].

Importantly, elements of successful eCR implementation were consistent across diverse health care delivery models, including academic medical centers, private hospitals, and public delivery systems across the United States. Our study is timely, given the widespread need and interest for systems that enhance the coordination and value of specialty care. The American College of Physicians’ Patient Centered Medical Home-Neighborhood framework for care delivery and the National Council on Quality Assurance’s Patient-Centered Specialty Practice Recognition program both promote specialty care coordination through tracking and coordination of referrals with the goals of enhanced communication and population management for specialty practices [21, 22]. eCR systems and other technologies that encourage knowledge sharing in new and efficient ways have great potential to advance these goals [23]. Highlighting this point is a $7 million Centers for Medicare and Medicaid Innovation grant recently awarded to the American Association of Medical Colleges to implement and evaluate eCR platforms in five U.S. academic medical centers [24].

Many questions regarding eCRs remain unanswered. Financial evaluation of eCR systems is nascent at this time. Data from our organization and others have demonstrated improved access to and efficiency of specialty care delivery with electronic consultation systems, with a shift from in-person visits to virtual co-management, which presumably decreases overall costs to the system [25, 26]. But, eCR systems increase PCP costs in the form of a greater burden to provide care that would have otherwise been provided by specialists and specialist costs in the form of time spent reviewing and responding to electronic consultations [15]. Robust measures of patient experience as well as of clinical impact on both individual patient outcomes and population health are needed.

Like all qualitative studies, there are limitations to our results. Interviews were conducted with only 1–2 leaders of each organization while eCR systems rely on support from many leaders in a health care system. Thus responses reflect the views and knowledge of those individuals and may not be representative of others in leadership roles. However, we believe that we identified the individuals most knowledgeable and responsible for eCR system implementation within each organization. After data analysis, we became aware of additional organizations that had implemented eCR systems, including some Veteran’s Administration and Kaiser Permanente sites [12, 15, 27]. Given the similar drivers, barriers and facilitators noted by leaders from diverse systems involved in various stages of eCR maturity (i.e., from pilot studies to full expansion), the themes presented in this analysis are likely applicable to those organizations as well.

## Conclusion

In summary, eCR systems have great potential to streamline access to and enhance the coordination and appropriateness of specialty care delivery. With several different eCR models from which to choose, health care leaders interested in implementing an electronic referral or consultation systems would be wise to begin with a clear understanding of their organization’s challenges and what problems they are seeking to address with an eCR. In addition, there are key institutional investments that are required for successful implementation, such as funding for program management and clinician incentives. As organizations gain greater experience with existing systems, more data will emerge with regards to key eCR platform functionality, costs, and clinical impact. Meanwhile, policy makers and payers should encourage ongoing development and evaluation of eCR systems. Clarification of the medico-legal implications of electronic consultation, for example, could mitigate one barrier to eCR implementation. Reimbursement of specialist reviewer effort and/or care coordination and financial support for the development of eCR software that can integrate with existing EMRs could also spur further innovation in this area of health system redesign.

## Additional file

Additional file 1:
**Table S1. **Key informant interview guide. **Table S2.** A priori analytic codes. **Table S3a**–**c.** Description, functionality, workflow, and implementation status of electronic referral, consultation and integrated eCR systems. (DOCX 128 kb)

## Abbreviations

eCR: electronic referral and consultation
EMR: electronic medical record
PCP: primary care provider
SFGH: San Francisco General Hospital

## References

[1] Cook NL, Hicks LS, O’Malley AJ, Keegan T, Guadagnoli E, Landon BE (2007). Access to specialty care and medical services in community health centers. Health Aff (Millwood).

[2] Forrest CB, Nutting PA, von Schrader S, Rohde C, Starfield B (2006). Primary care physician specialty referral decision making: patient, physician, and health care system determinants. Med Decis Making.

[3] Felt-Lisk S, McHugh M, Howell E (2002). Monitoring local safety-net providers: do they have adequate capacity?. Health Aff (Millwood).

[4] Singer SJ, Reyes Nieva H, Brede N, Ling J, Leydon N, Weissman JS (2015). Evaluating ambulatory practice safety: the PROMISES project administrators and practice staff surveys. Med Care.

[5] Gandhi TK, Sittig DF, Franklin M, Sussman AJ, Fairchild DG, Bates DW (2000). Communication breakdown in the outpatient referral process. J Gen Intern Med.

[6] Kim Y, Chen AH, Keith E, Yee HF, Kushel MB (2009). Not perfect, but better: primary care providers’ experiences with electronic referrals in a safety net health system. J Gen Intern Med.

[7] Kim-Hwang JE, Chen AH, Bell DS, Guzman D, Yee HF, Kushel MB (2010). Evaluating electronic referrals for specialty care at a public hospital. J Gen Intern Med.

[8] Warren J, White S, Day KJ, Gu Y, Pollock M (2011). Introduction of electronic referral from community associated with more timely review by secondary services. Appl Clin Inform.

[9] Liddy C, Maranger J, Afkham A, Keely E (2013). Ten steps to establishing an e-consultation service to improve access to specialist care. Telemed J E Health.

[10] Vimalananda VG, Gupte G, Seraj SM, Orlander J, Berlowitz D, Fincke BG (2015). Electronic consultations (e-consults) to improve access to specialty care: A systematic review and narrative synthesis. J Telemed Telecare.

[11] 111 United States Congress: Patient Protection and Affordable Care Act.; 2010.

[12] Horner K, Wagner EH, Tufano J: Electronic Consultations between primary and specialty care clinicians: early insights. The Commonwealth Fund Issue Brief; October, 2011. 22059281

[13] Hsieh HF, Shannon SE (2005). Three approaches to qualitative content analysis. Qual Health Res.

[14] Tuot DS, Murphy EJ, McCulloch CE, Leeds K, Chan E, Chen AH: Leveraging an electronic referral and consultation system to provide high quality corrindated specialty care. Health Care: The Journal of Delivery Science and Innovation In press.

[15] McAdams M, Cannavo L, Orlander JD (2014). A Medical Specialty e-Consult Program in a VA Health Care System. Fed. Pract..

[16] Palen TE, Price D, Shetterly S, Wallace KB (2012). Comparing virtual consults to traditional consults using an electronic health record: an observational case–control study. BMC Med Inform Decis Mak.

[17] McGinn CA, Grenier S, Duplantie J, Shaw N, Sicotte C, Mathieu L (2011). Comparison of user groups’ perspectives of barriers and facilitators to implementing electronic health records: a systematic review. BMC Med.

[18] Scholle SH, Asche SE, Morton S, Solberg LI, Tirodkar MA, Jaen CR (2013). Support and strategies for change among small patient-centered medical home practices. Ann Fam Med.

[19] Wan TT, Demachkie Masri M, Ortiz J, Lin BY (2014). Willingness to participate in accountable care organizations: health care managers’ perspective. Health Care Manag (Frederick).

[20] Carrier E, Dowling MK, Pham HH (2012). Care coordination agreements: barriers, facilitators, and lessons learned. Am J Manag Care.

[21] Laine C (2011). Welcome to the patient-centered medical neighborhood. Ann Intern Med.

[22] National Committee for Quality Assurance. [http://www.ncqa.org/Programs/Recognition/Practices/PatientCenteredSpecialtyPracticePCSP.aspx]

[23] Kirsh SR, Ho PM, Aron DC (2014). Providing specialty consultant expertise to primary care: an expanding spectrum of modalities. Mayo Clin Proc.

[24] Davis A, Gilchrist V, Grumbach K, James P, Kallenberg R, Shipman SA (2015). Advancing the Primary/Specialty Care Interface through Econsults and Enhanced Referrals. Ann Fam Med.

[25] Chen AH, Murphy EJ, Yee HF (2013). eReferral--a new model for integrated care. N Engl J Med.

[26] Liddy C, Rowan MS, Afkham A, Maranger J, Keely E (2013). Building Access to specialist care through e-consultation. Open Med.

[27] North F, Uthke LD, Tulledge-Scheitel SM (2014). Integration of e-consultations into the outpatient care process at a tertiary medical centre. J Telemed Telecare.

