# Supplementary material for: Facilitators and barriers to implementing electronic referral and/or consultation systems: a qualitative study of 16 health organizations
Source: BMC Health Serv Res. 2015 Dec 19;15:568. doi: 10.1186/s12913-015-1233-1 (PMC4684927; doi:10.1186/s12913-015-1233-1)
Supplement: Additional file 1: — Table S1. Key informant interview guide. Table S2. A priori analytic codes. Table S3a–c. Description, functionality, workflow, and implementation status of electronic referral, consultation and integrated eCR systems. (DOCX 128 kb) [file 12913_2015_1233_MOESM1_ESM.docx]

| **Additional Table 1: Key informant interview guide** | |
| --- | --- |
| 1. | Please describe your health delivery system:   - Where is your organization located? - What type of organization? (Academic medical center, community hospital, health plan, integrated delivery system, public system, public hospital, tertiary or secondary care hospital - How many patients are served annually? - What are the types and percentages of insurers? |
| 2. | Please describe your health delivery system’s drivers for implementing an electronic referral system. |
| 3. | Was the electronic referral/consultation system purchased or home-grown? |
| 4. | Who uses the electronic referral/consultation system? |
| 5. | Please describe the facilitators for implementing the system. |
| 6. | Please describe the barriers encountered to implementing the system. |
| 7. | Please describe evaluation metrics your health delivery system currently tracks related to your electronic referral system. |
| 8. | Please describe your organization’s best practices, and how are those best practices spread throughout the organization? |
| 9. | What key lessons learned can be shared with other organizations? |

| **Additional Table 3a: Description, functionality, workflow, and implementation status of electronic REFERRAL systems*** | | | | | |
| --- | --- | --- | --- | --- | --- |
| **Type of Organization** | **Description of electronic referral system** | **Functionality** | **Workflow** | **Implementation status, including specialties involved** | **EMR status, EMR vendor** |
| **Health Plan** | Pilot electronic referral management system with five specialties and two primary care sites for commonly referred conditions. | ☐Integrated with EMR  ☒Upload reports/studies  ☒Referral guidelines and/or minimum datasets  ☒Referral management/tracking system  ☐Scheduling capability  ☐Bidirectional communication  ☒Accessible by external providers  ☐Accessible by patients  ☒ Referral template (vs. text box for free text) | ☒ Referring provider electronically submits referral  ☐ Referral manager electronic submits referral  ☐ Referring provider can choose specialist reviewer  ☐ Designated specialist reviewers who receive all communication  ☒ Administrative staff or referral coordinators triage referral requests and distribute to appropriate specialist  ☒ Specialist provides recommendations (schedule, not-schedule with recommendations)  ☒ Specialist sends scheduled referrals to a scheduler or patient call center | Pilot: July – November 2013 with 4 medical specialties and 1 diagnostic specialty | Multiple |
| **Community Health Center - Network** | Electronic referral management system is used to refer patients to nine specialties, whose volunteers have clinic hours dispersed among four sites. Staff have customized the referral template within its electronic medical record. | ☒Integrated with EMR  ☐Upload reports/studies  ☒Referral guidelines and/or minimum datasets  ☒Referral management/tracking system  ☐Scheduling capability  ☐Bidirectional communication  ☐Accessible by external providers  ☐Accessible by patients  ☒ Referral template (vs. text box for free text) | ☒ Referring provider electronically submits referral  ☐ Referral manager electronic submits referral  ☐ Referring provider can choose specialist reviewer  ☐ Designated specialist reviewers who receive all communication  ☒ Administrative staff or referral coordinators triage referral requests and distribute to appropriate specialist  ☐ Specialist provides recommendations (schedule, not-schedule with recommendations)  ☒ Specialist sends scheduled referrals to a scheduler or patient call center | Implemented: 2010 - 2012 with 4 medical specialties, 3 surgical specialties, 2 rehabilitation services, | NextGen |
| **Public system - county** | A legacy, homegrown system is used for referral tracking and billing. NextGen is being implemented, and will be used to manage referrals from hospital clinics. | ☒Integrated with EMR  ☐Upload reports/studies  ☒Referral guidelines and/or minimum datasets  ☒Referral management/tracking system  ☐Scheduling capability  ☐Bidirectional communication  ☒Accessible by external providers  ☐Accessible by patients  ☐ Referral template (vs. text box for free text) | ☒ Referring provider electronically submits referral  ☐ Referral manager electronic submits referral  ☐ Referring provider can choose specialist reviewer  ☐ Designated specialist reviewers who receive all communication  ☒ Administrative staff or referral coordinators triage referral requests and distribute to appropriate specialist  ☐ Specialist provides recommendations (schedule, not-schedule with recommendations)  ☐ Specialist sends scheduled referrals to a scheduler or patient call center | Implemented: HCRM  Pre-pilot: NextGen  Pre-pilot: i2iTracks | Homegrown system: Health CRM  NextGen implementation began in March 2014 |
| **Academic Medical Center** | This is a hybrid phone/web/fax referral system used by internal and external referring providers. | ☐Integrated with EMR  ☒Upload reports/studies  ☐Referral guidelines and/or minimum datasets  ☒Referral management/tracking system  ☐Scheduling capability  ☐Bidirectional communication  ☒Accessible by external providers  ☒Accessible by patients  ☐ Referral template (vs. text box for free text) | ☒ Referring provider electronically submits referral  ☐ Referral manager electronic submits referral  ☐ Referring provider can choose specialist reviewer  ☐ Designated specialist reviewers who receive all communication  ☒ Administrative staff or referral coordinators triage referral requests and distribute to appropriate specialist  ☐ Specialist provides recommendations (schedule, not-schedule with recommendations)  ☒ Specialist sends scheduled referrals to a scheduler or patient call center | Pilot: Referral Center: spring 2012 for all services  Implemented for internal referring providers in July 2013 for all services | AllScripts and DbMotion |
| **Public system - county** | A web-based system developed by Buena Digital, is used primarily for data management. It is being integrated with two electronic medical records, Cerner and NextGen, which are used by the County and community clinics, respectively. | ☒Integrated with EMR  ☐Upload reports/studies  ☐Referral guidelines and/or minimum datasets  ☒Referral management/tracking system  ☐Scheduling capability  ☐Bidirectional communication  ☐Accessible by external providers  ☐Accessible by patients  ☐ Referral template (vs. text box for free text) | ☒ Referring provider electronically submits referral  ☐ Referral manager electronic submits referral  ☐ Referring provider can choose specialist reviewer  ☐ Designated specialist reviewers who receive all communication  ☒ Administrative staff or referral coordinators triage referral requests and distribute to appropriate specialist  ☐ Specialist provides recommendations (schedule, not-schedule with recommendations)  ☒ Specialist sends scheduled referrals to a scheduler or patient call center | Implemented: 2007 – present with 17 specialties presently | Cerner |

* Checked boxes depict active functionality and workflow present for each organization’s electronic referral system.

| **Additional Table 3b: Description, functionality, workflow, and implementation status of electronic CONSULTATION systems*** | | | | | |
| --- | --- | --- | --- | --- | --- |
| **Type of Organization** | **Description of electronic referral system** | **Functionality** | **Workflow** | **Implementation status, including specialties involved** | **EMR status, EMR vendor** |
| **Non-profit organzation** | Piloted eConsult, developed by NetChemistry, by 20 unique community clinics in 2012 to refer to medical specialties, complementing the agency’s mission of providing access to surgical care for uninsured patients. | ☐Integrated with EMR  ☒Upload reports/studies  ☐Referral guidelines and/or minimum datasets  ☒Referral management/tracking system  ☐Scheduling capability  ☒Bidirectional communication  ☐Accessible by external providers  ☐Accessible by patients  ☐ Referral template (vs. text box for free text) | ☒ Referring provider electronically submits referral  ☐ Referral manager electronic submits referral  ☐ Referring provider can choose specialist reviewer  ☒ Designated specialist reviewers who receive all communication  ☐ Administrative staff or referral coordinators triage referral requests and distribute to appropriate specialist  ☒ Specialist provides recommendations (schedule, not-schedule with pure electronic consultation)  ☒ Specialist sends scheduled referrals to a scheduler or patient call center | Pilot: 2012  250 patients in pilot; no longer active  25 medical and surgical specialties | Not applicable |
| **Public health system - county** | RefTrak is a web-based referral submission and tracking system used by independent community clinics.  Hospital-based providers submit specialty clinic referrals through their EMR. | ☒Integrated with EMR  ☒Upload reports/studies  ☒Referral guidelines and/or minimum datasets  ☒Referral management/tracking system  ☐Scheduling capability  ☒Bidirectional communication  ☒Accessible by external providers  ☐Accessible by patients  ☐ Referral template (vs. text box for free text) | ☐ Referring provider electronically submits referral  ☒ Referral manager electronic submits referral  ☐ Referring provider can choose specialist reviewer  ☐ Designated specialist reviewers who receive all communication  ☒ Administrative staff or referral coordinators triage referral requests and distribute to appropriate specialist  ☒ Specialist provides recommendations (schedule, not-schedule with pure electronic consultation)  ☐ Specialist sends scheduled referrals to a scheduler or patient call center | Implemented: 2003, nearly all specialties receive referrals electronically | Community clinics: NextGen  Hospital: Siemens Soarian |
| **Academic Medical Center** | Piloting a web-based Referral Portal (par8to) and Pre-consultative exchange system that will be integrated with the organization’s new electronic medical record in 2014-15. | ☐Integrated with EMR (planned)  ☒Upload reports/studies  ☐Referral guidelines and/or minimum datasets  ☒Referral management/tracking system  ☐Scheduling capability  ☒Bidirectional communication  ☐Accessible by external providers  ☐Accessible by patients  ☒ Referral template (vs. text box for free text) | ☒ Referring provider electronically submits referral  ☐ Referral manager electronic submits referral  ☐ Referring provider can choose specialist reviewer  ☒ Designated specialist reviewers who receive all communication  ☒ Administrative staff or referral coordinators triage referral requests and distribute to appropriate specialist  ☒ Specialist provides recommendations (schedule, not-schedule with pure electronic consultation)  ☒ Specialist sends scheduled referrals to a scheduler or patient call center | Pilot: 2013 with 7 medical specialties  Implementation phase: planned for 2014 with all 70-80 medical and surgical specialties | Current: Homegrown system  2014-15: Implementing Epic |
| **Public system - county** | Providers currently use the clinical messaging tool built into the electronic medical record (eClinicalWorks P2P).  Smart Referral System (planned, vendor identification is in progress). | ☒Integrated with EMR  ☒Upload reports/studies  ☐Referral guidelines and/or minimum datasets]  ☒Referral management/tracking system  ☐Scheduling capability  ☒Bidirectional communication  ☐Accessible by external providers  ☐Accessible by patients  ☐ Referral template (vs. text box for free text) | ☒ Referring provider electronically submits referral  ☐ Referral manager electronic submits referral  ☒ Referring provider can choose specialist reviewer  ☐ Designated specialist reviewers who receive all communication  ☐ Administrative staff or referral coordinators triage referral requests and distribute to appropriate specialist  ☒ Specialist provides recommendations (schedule, not-schedule with pure electronic consultation)  ☒ Specialist sends scheduled referrals to a scheduler or patient call center | Pre-pilot: Smart Referral System  Implemented: electronic consultations | eClinicalWorks |
| **Academic medical center** | Web-based electronic referral system to connect referring providers at other health delivery systems to specialists at no extra cost.  This hospital is implementing Epic and will develop a new electronic consultation system embedded into the new EMR. | ☐Integrated with EMR  ☒Upload reports/studies  ☐[Referral guidelines] and/or minimum datasets  ☒Referral management/tracking system  ☐Scheduling capability  ☒Bidirectional communication  ☒Accessible by external providers  ☐Accessible by patients  ☒ Referral template (vs. text box for free text) | ☒ Referring provider electronically submits referral  ☐ Referral manager electronic submits referral  ☐ Referring provider can choose specialist reviewer  ☒ Designated specialist reviewers who receive all communication  ☐ Administrative staff or referral coordinators triage referral requests and distribute to appropriate specialist  ☒ Specialist provides recommendations (schedule, not-schedule with pure electronic consultation)  ☒ Specialist sends scheduled referrals to a scheduler or patient call center | Implemented: October 2011 – present with 39 medical and surgical specialties | Epic |
| **Academic Medical Center** | EMR-based structured electronic referral forms (eReferral) and electronic consultation (eConsult) forms for submitting consultative questions; these two tools are integrated into Epic, its electronic medical record. | ☒Integrated with EMR  ☐Upload reports/studies  ☒Referral guidelines and/or minimum datasets  ☒Referral management/tracking system  ☐Scheduling capability  ☒Bidirectional communication  ☐Accessible by external providers  ☐Accessible by patients  ☒ Referral template (vs. text box for free text) | ☒ Referring provider electronically submits referral  ☐ Referral manager electronic submits referral  ☐ Referring provider can choose specialist reviewer  ☒ Designated specialist reviewers who receive all communication  ☒ Administrative staff or referral coordinators triage referral requests and distribute to appropriate specialist  ☒ Specialist provides recommendations (schedule, not-schedule with pure electronic consultation)  ☒ Specialist sends scheduled referrals to a scheduler or patient call center | Implemented eReferral in April 2012 with 12 medical specialties  Implemented eConsult in September 2012 with 12 medical specialties | Epic |

* Checked boxes depict active functionality and workflow present for each organization’s electronic consultation system.

* Functionalities in [parentheses] indicate planned functionality that have not yet been implemented.

| **Additional Table 3c: Description, functionality, workflow, and implementation status of INTEGRATED eCR systems*. San Francisco Health Network included as a reference.** | | | | | |
| --- | --- | --- | --- | --- | --- |
| **Type of Organization** | **Description of electronic referral system** | **Functionality** | **Workflow** | **Implementation status, including specialties involved** | **EMR status, EMR vendor** |
| **San Francisco Health Network** | eReferral is the sole referral system for San Francisco’s publically funded, vertically-integrated health care delivery system. All specialists, including reviewers, are based at the San Francisco General Hospital. | ☒Integrated with EMR  ☐Upload reports/studies  ☒Referral guidelines and/or minimum datasets  ☒Referral management/tracking system  ☐Scheduling capability  ☒Bidirectional communication  ☒Accessible by external providers  ☐Accessible by patients  ☐ Referral template (vs. text box for free text) | ☒ Referring provider electronically submits referral  ☒ Referral manager electronic submits referral  ☐ Referring provider can choose specialist reviewer  ☒ Designated specialist reviewers who receive all communication  ☐ Administrative staff or referral coordinators triage referral requests and distribute to appropriate specialist  ☒ Specialist provides recommendations (schedule, not-scheduled with virtual co-management)  ☒ Specialist sends scheduled referrals to a scheduler or patient call center | Implemented: 2009 with over 50 medical and surgical specialties and diagnostic services | Homegrown system |
| **Community health center - network** | The pilot eCR partnered with a Academic Medical Centers for referring to cardiology and pain management, using the electronic messaging system built into the electronic medical record (eClinicalWorks P2P module). | ☒Integrated with EMR  ☒Upload reports/studies  ☐Referral guidelines and/or minimum datasets  ☒Referral management/tracking system  ☐Scheduling capability  ☒Bidirectional communication  ☒Accessible by external providers  ☐Accessible by patients  ☐ Referral template (vs. text box for free text) | ☒ Referring provider electronically submits referral  ☐ Referral manager electronic submits referral  ☐ Referring provider can choose specialist reviewer  ☒ Designated specialist reviewers who receive all communication  ☐ Administrative staff or referral coordinators triage referral requests and distribute to appropriate specialist  ☒ Specialist provides recommendations (schedule, not-scheduled with virtual co-management)  ☐ Specialist sends scheduled referrals to a scheduler or patient call center | Implemented:  2 medical specialties | eClinicalWorks |
| **Health plan** | This vendor web-based electronic consultation system (NetChemstiry) was developed in collaboration with a number physician groups that serve over 700,000 patients. | ☐[Integrated with EMR]  ☒Upload reports/studies  ☒Referral guidelines and/or minimum datasets  ☒Consult management/ tracking system  ☐Scheduling capability  ☒Bidirectional communication  ☒Accessible by external providers  ☐Accessible by patients  ☒ Referral template (vs. text box for free text) | ☒ Primary care provider electronically initiates consult to Specialist Reviewer  ☐ Referral manager electronic submits referral  ☐ Referring provider can choose specialist reviewer  ☒ Designated specialist reviewers who receive all communication  ☐ Administrative staff or referral coordinators triage referral requests and distribute to appropriate specialist  ☒ Specialist provides recommendations (schedule, not-schedule with pure electronic consultation)  ☐ Specialist sends scheduled referrals to a scheduler or patient call center | Implemented:  30+ medical and surgical specialties; 4,000 users; 194 sites; 700,000 patients | N/A |
| **Public health system - county** | This is a vendor web-based electronic consultation system (NetChemstiry) that was developed in collaboration with a health plan. | ☐[Integrated with EMR]  ☒Upload reports/studies  ☒Referral guidelines and/or minimum datasets  ☒Referral management/tracking system  ☐Scheduling capability  ☒Bidirectional communication  ☒Accessible by external providers  ☐Accessible by patients  ☒ Referral template (vs. text box for free text) | ☒ Referring provider electronically submits referral  ☐ Referral manager electronic submits referral  ☐ Referring provider can choose specialist reviewer  ☒ Designated specialist reviewers who receive all communication  ☐ Administrative staff or referral coordinators triage referral requests and distribute to appropriate specialist  ☒ Specialist provides recommendations (schedule, not-schedule with pure electronic consultation)  ☒ Specialist sends scheduled referrals to a scheduler or patient call center | Implemented:  23 medical and surgical specialties | Current: legacy system called Affinity  2014-2015: implementing Cerner |

* Checked boxes depict active functionality and workflow present for each organization’s electronic consultation system.

* Functionalities in [parentheses] indicate planned referral system functionality that have not yet been implemented.
